# Thermoelectric Nanocomposites and Segmented Single-Leg Device Based on GeTe and (Bi,Sb)_2_Te_3_

**DOI:** 10.3390/ma19071345

**Published:** 2026-03-28

**Authors:** Lawrence Yongo Methodius Emiliano, Yilin Jiang, Hua-Lu Zhuang, Hezhang Li, Chen Chen, Su-Wei Zhang, Yuzuru Miyazaki, Jing-Feng Li

**Affiliations:** 1 State Key Laboratory of New Ceramic Materials, School of Materials Science and Engineering, Tsinghua University, Beijing 100084, China; lawrenceyongo150@gmail.com (L.Y.M.E.); jyl_materials@163.com (Y.J.); hualu@szu.edu.cn (H.-L.Z.); lihezhang1202@mail.tsinghua.edu.cn (H.L.); c-chen@tsinghua.edu.cn (C.C.); zhangsw999@tsinghua.edu.cn (S.-W.Z.); 2 Department of Applied Physics, Graduate School of Engineering, Tohoku University, Sendai 980-8579, Japan; yuzuru.miyazaki.b7@tohoku.ac.jp

**Keywords:** thermoelectric, GeTe, (Bi,Sb)_2_Te_3_, nanocomposites, segmented device, conversion efficiency

## Abstract

Thermoelectric (TE) materials offer a promising route for direct thermal-to-electrical energy conversion via the Seebeck effect. Among them, GeTe exhibits superior performance in the mid-temperature range (500–800 K), whereas (Bi,Sb)_2_Te_3_ is widely regarded as the benchmark material for near low-temperature applications (< 450 K). To improve TE efficiency over a wider temperature range, segmented GeTe/(Bi,Sb)_2_Te_3_-based single-leg TE devices were developed. Specifically, based on nanocomposite technology, B_4_C and SiC nanoparticles were, respectively, introduced into GeTe and (Bi,Sb)_2_Te_3_, achieving optimization of electrical conductivity alongside reduction in thermal conductivity, thereby enhancing the thermoelectric figure of merit (*ZT*). Finite element simulations were used to optimize the geometric structure of the segmented device, determining the ideal ratio of GeTe to (Bi,Sb)_2_Te_3_. The simulations predicted a maximum conversion efficiency (*η*_max_) of 16.9% when the ratio of GeTe to (Bi,Sb)_2_Te_3_ was 0.24, with a power density of 18.5 mW/mm^2^. Experimentally, the fabricated segmented device attained a peak conversion efficiency of 7.14% and a power density of 12.5 mW/mm^2^ under a hot-side temperature of 773 K. These findings confirm that strategic segmentation, combined with nanoscale phonon scattering engineering, substantially improves overall TE device performance across broad temperature range, underscoring its potential for high-efficiency thermoelectric energy conversion systems.

## 1. Introduction

Thermoelectric (TE) materials, known for enabling direct conversion of heat into electricity and vice versa, have attracted significant interest in recent years due to their potential for sustainable energy solutions, including power generation and solid-state cooling [1]. TE technology, along with solid-state devices that exploit TE effect, offers several advantages, including having no moving parts or refrigerants, coupled with high reliability and scalability, marking a substantial improvement over conventional methods [2]. The technology has thus sparked global interest across various sectors, including applications such as waste heat recovery and harnessing solar thermal energy, portable picnic coolers, and thermal management in microprocessors [3,4].

For a TE device, the maximum efficiency (*η*_max_) in converting thermal energy to electrical energy is determined by both the Carnot efficiency, operating temperatures, and the TE material’s dimensionless figure of merit (*ZT*) [5], given by the equation:

(1) $$\eta_{max}=\frac{T_{h}-T_{c}\;\sqrt{1+Z\frac{T_{h}+T_{c}}{2}\;}-1}{T_{h}\;\sqrt{1+Z\frac{T_{h}+T_{c}}{2}}+\frac{T_{c}}{T_{h}}}$$

where *T*_h_ is the hot-side temperature, *T_c_* is the cold-side temperature, and *Z*(*T*_h_ + *T*_c_)/2 is the average TE figure of merit. The performance of TE device is dominated by *ZT*, which is defined as: *ZT* = *σ*·*S*^2^*T*/*κ*_tot_ where *S* is the Seebeck coefficient, *σ* is the electrical conductivity, *κ*_tot_ is the total thermal conductivity, *T* is the absolute temperature and the product (*σ*·*S*^2^), is the power factor (*PF*) [6]. Moreover, the total thermal conductivity, (*κ*_tot_), is composed of the lattice thermal conductivity (*κ*_lat_) and the electronic thermal conductivity (*κ*_ele_). To improve the efficiency of TE devices, i.e., maximize its *ZT*, it is essential to increase the *PF* while simultaneously decreasing the *κ*_lat_. Independently manipulating these three variables (*σ*, *S*, *κ*_tot_) is a crucial strategy to achieving a higher *ZT* [6].

Recent progress in the field of TE materials research has been substantial, driven by the development of innovative processes and novel concepts. A set of newly discovered TE materials has demonstrated excellent TE performance [7,8,9,10,11]. Currently, the most advanced TE materials are studied for their capabilities in power generation applications and have demonstrated high TE performance, including Bi_2_Te_3_, half-Heusler, GeTe, PbTe, and others [12,13,14,15,16,17].

Studies on (Bi,Sb)_2_Te_3_-based TE materials have achieved *ZT* values from 1.4 to 1.6 at near room-temperatures (300–500 K) through the strategic manipulation of point defects and dislocation engineering [18,19,20,21]. Furthermore, research into selenium-free n-type solid solutions of Bi_2−*x*_Sb*_x_*Te_3_ has demonstrated the feasibility of achieving competitive properties using a two-step process of mechanical alloying (MA) followed by SPS, which facilitates the precise control of stoichiometry and microstructure [22]. Similarly, for GeTe-based alloys, optimizing carrier concentration and reducing lattice thermal conductivity has allowed *ZT* values to reach 1.8 to 2.0 or higher [23,24]. For instance, Jiang, Y et al. achieved a maximum *ZT* > 2.3 at 648 K for a lead-free GeTe-based Bi_0.07_Ge_0.90_Te compound, synthesized using MA combined SPS [25].

Numerous materials incorporating nanoparticles have demonstrated excellent performance due to intensified carrier and phonon scattering [26]. Wu et al. enhanced p-type Bi_0.42_Sb_1.58_Te_3_ by incorporating Ag_5_SbSe_4_, resulting in a peak *ZT* of 1.40 and a conversion efficiency of 6.5% at Δ*T* = 200 K [27]. Additionally, Li et al. introduced superparamagnetic Fe_3_O_4_ nanoparticles into a p-type Bi_0.5_Sb_1.5_Te_3_ matrix, yielding a 32% improvement in *ZT* (1.5 at 340 K) by simultaneously enhancing the Seebeck coefficient and reducing thermal conductivity [28]. Building on these principles, the current study utilizes B_4_C and SiC nanoparticles to achieve synergistic modulation of transport properties. B_4_C is employed because the thermal mismatch between the boron-based particles and the matrix induces high-density dislocations that effectively scatter mid-frequency phonons, significantly reducing lattice thermal conductivity [29]. Furthermore, the introduction of SiC as a secondary phase is highly effective for decoupling interdependent TE parameters; its high-temperature stability and ability to form dense nanostructured grain boundaries facilitate intensified phonon scattering without severely compromising carrier mobility [30]. This success in nanocomposite engineering inspired the current study to further enhance the TE performance of GeTe and (Bi,Sb)_2_Te_3_ through the targeted introduction of nanoparticles.

Several TE devices utilizing GeTe-based materials have been developed with favorable efficiencies [31,32,33]. For example, Jia et al. reported a maximum conversion efficiency (*η*_max_) of 11.8% for a GeTe single-leg device at Δ*T* = 422 K [34]. To bridge the efficiency gap across wider temperature range, segmented architectures are employed. Pei et al. fabricated a segmented GeTe/(Bi,Sb)_2_Te_3_ single-leg device, achieving *η*_max_ = 9.5% and a power density of ≈7.45 mWmm^−2^ [35]. Furthermore, Cao et al. reported a record *η*_max_ = 13.6% in a Bi_0.5_Sb_1.5_Te_3_/Ge_0.9_Sb_0.1_Te segmented single-leg device at Δ*T* = 450 K [36]. While the peak efficiency in this work is lower than these benchmarks, our study demonstrates a competitive output power density and introduces a more scalable one-step consolidation process using B_4_C and SiC nanocomposites to maintain performance over broad thermal gradients.

The metallization layer is a critical factor in the fabrication of these segmented devices, as it minimizes contact resistance and ensures mechanical integrity [37]. This reliability is paramount for practical applications, as chemical reaction and atomic diffusion at elevated temperatures are primary factors in device failure. Fe, Ni, and Ti are commonly utilized; specifically, Ni is a standard metallization layer for (Bi,Sb)_2_Te_3_ due to its low contact resistance (<1 µΩ∙cm^2^) and high bonding strength (~30 MPa) [37]. Beyond individual metals, advanced engineering roadmaps now utilize density functional theory (DFT) to screen for reliable semimetallic interface materials that suppress diffusion and match thermal expansion coefficients [38]. Furthermore, transition metal silicides like FeSi have been demonstrated as potent barrier layers that can be integrated via a one-step consolidation process to achieve low contact resistivities (~20 µΩ∙cm^2^) and stable conversion efficiencies [39]. For GeTe segments, Ti is preferred as an effective metallization layer due to its compatible linear expansivity (10.80 × 10^−6^ K^−1^), low contact resistance (3 μΩ cm^2^), and minimal diffusion layer thickness (0.4 µm) [32]. The strategic combination of these optimized nanocomposites into a segmented single-leg device allows for the effective utilization of material strengths across a broad thermal gradient.

This study leverages the high performance of GeTe- and (Bi,Sb)_2_Te_3_-based nanocomposites to fabricate a segmented single-leg thermoelectric device optimized for operation across a broad temperature range. By incorporating 0.6 wt.% B_4_C and 0.4 vol.% SiC as reinforcing nanoparticles, *p*-type Bi_0.05_Ge_0.99_Te and Bi_0.4_Sb_1.6_Te_3_ achieved peak *ZT* values of 2.2 at 723 K and 1.3 at 348 K, respectively. To translate these material-level advances into device-level performance, finite element analysis was employed to optimize the device geometry, establishing a segment cross-sectional area ratio of 0.24. The *p*-type segments were joined using a specialized metallization layer and consolidated via a one-step spark plasma sintering (SPS) process. While simulations predicted a maximum conversion efficiency (*η*_max_) of 16.9%, the fabricated device experimentally achieved a peak *η*_max_ of 7.14% and a power density of 12.5 mW/mm^2^ at a hot-side temperature of 773 K. These results validate the synergy between nanocomposite engineering and strategic segmentation for efficient thermoelectric energy conversion.

The primary novelty of this work lies in the synergistic application of distinct nanoparticle reinforcements, B_4_C and SiC to independently optimize the thermoelectric performance of the mid-temperature (GeTe) and low-temperature ((Bi,Sb)_2_Te_3_) segments, respectively. Unlike previous reports on segmented devices that have primarily focused on stoichiometric tuning, our approach employs dual-nanocomposite engineering to achieve record peak *ZT* values of 2.2 in GeTe and 1.3 in (Bi,Sb)_2_Te_3_ within a single integrated device architecture. Furthermore, this study establishes a comprehensive materials-to-device framework that integrates three-dimensional finite element simulation for geometric optimization with a streamlined one-step SPS fabrication process. This integrated strategy not only yields a robust segmented single-leg device with a competitive power density of 12.5 mW/mm^2^ but also demonstrates a practical and scalable pathway for high-performance waste heat recovery applications.

## 2. Materials and Methods

Nanocomposite materials with the nominal compositions Bi_0.05_Ge_0.99_Te + *x* wt.% B_4_C (*x* = 0, 0.1, 0.4, 0.6, and 1.0) and Bi_0.4_Sb_1.6_Te_3_ + *x* vol.% SiC (*x* = 0.2, 0.4, 0.6, and 0.8) were synthesized via a two-step process comprising mechanical alloying (MA) and spark plasma sintering (SPS). High-purity elemental powders of Ge (99.999%, Zhongnuo New Materials Technology Co., Ltd., Beijing, China), Te (99.999%, Zhongnuo New Materials Technology Co., Ltd.), Bi (99.99%, Shanghai Aladdin Biochemical Technology Co., Ltd., Shanghai, China), Sb (99.99%, Shanghai Aladdin Biochemical Technology Co., Ltd.), B_4_C (99%, Aladdin Industrial Corporation Co., Ltd.), and SiC (99%, Beijing Jinming Biotechnolgy Co., Ltd., Beijing, China) served as starting materials. The main properties of the raw materials are summarized in the (Table S1). For the GeTe-based alloys, MA was conducted in a tungsten carbide milling jar to withstand the inherent hardness of Ge, whereas a hardened stainless-steel vial and ball set were employed for the (Bi,Sb)_2_Te_3_ series.

The MA process was executed using a planetary ball mill at rotation speeds of 450 rpm for 12 h (GeTe) and 480 rpm for 6 h ((Bi,Sb)_2_Te_3_). To prevent oxidation, milling was performed under a protective atmosphere of 95 vol.% Ar and 5 vol.% H_2_. The resulting precursor powders were consolidated into dense bulk specimens using an SPS system (Graphite die: ϕ 12 mm × 5 mm). GeTe-based samples were sintered at 723 K for 5 min under 60 MPa of axial pressure, while (Bi,Sb)_2_Te_3_-based samples were processed at 673 K for 5 min under 50 MPa. The thermoelectric properties and segmented device performance reported in this study are based on the characterization of a representative single sample for each composition and a single-leg device.

The Seebeck coefficient (*S*) and electrical resistivity (*ρ*) were measured simultaneously using a ZEM-3 system (Ulvac-Riko, Yokohama, Japan) on rectangular specimens (2.5 mm × 2.5 mm × 9 mm^3^) for GeTe; (2 mm × 2 mm × 8 mm^3^) for (Bi,Sb)_2_Te_3_ over their respective temperature range of 300–723 K and 300–473 K. Thermal conductivity (*κ*) was determined through the relation *κ = DC_P_d*, where D is the thermal diffusivity, *C*_P_ is the specific heat capacity, and d is the density. Diffusivity was measured on ϕ 6 mm × 1 mm disks using the laser flash method (LFA 457, NETZSCH, Selb, Germany). *C*_P_ was estimated via the Dulong-Petit limit, and density was measured using Archimedes’ method.

Microstructural analysis and diffusion layer investigations were performed using field-emission scanning electron microscopy (FESEM, Zeiss Merlin, Oberkochen, Germany), Electron Probe Microanalysis (EPMA) and Energy Dispersive X-ray Spectroscopy (EDS), and X-ray diffraction (XRD). The performance of the integrated segmented device, including output voltage (*U*), output power (*P*), and cooling capacity(*Q*_C_), was evaluated using a Mini-PEM system (Advance Riko, Yokohama, Japan). Energy conversion efficiency (*η*) was calculated as *η* = *P/(P + Q_C_)* × 100%. The segmented device’s ideal performance was simulated using high-fidelity 3D finite element modeling (COMSOL Multiphysics 5.4). The 3D finite element simulations were performed by using the experimentally measured temperature-dependent transport properties of the GeTe and (Bi,Sb)_2_Te_3_ nanocomposites as the governing material inputs. The simulation model assumes steady-state heat flow and ideal thermal/electrical insulation at the boundaries. At a hot-side temperature, the model predicted an optimal power density that follows the same parabolic trend as our experimental measurements.

## 3. Results and Discussion

**Figure 1:**
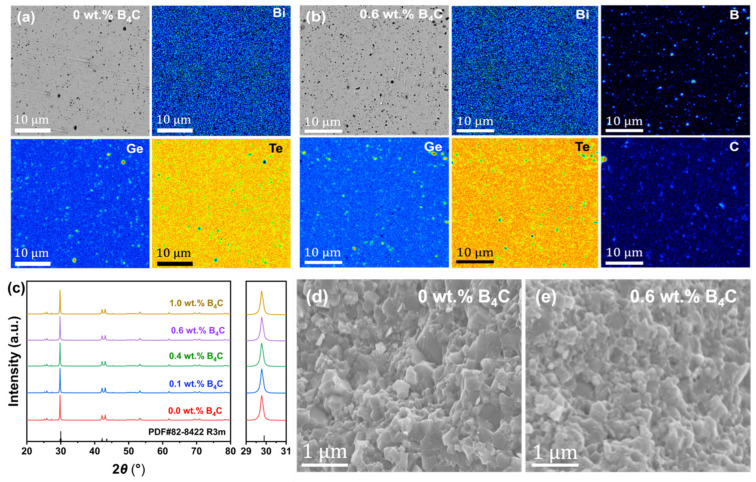
Microstructural and phase characterization of GeTe-based nanocomposites: (**a**,**b**) EPMA micrograph and EDS mapping of the polished surface of (**a**) Bi_0.05_Ge_0.99_Te and (**b**) Bi_0.05_Ge_0.99_Te + 0.6 wt.% B_4_C samples; (**c**) XRD patterns of Bi_0.05_Ge_0.99_Te + *x* wt.% B_4_C nanocomposites (*x* = 0, 0.1, 0.4, 0.6, and 1.0); (**d**,**e**) SEM micrographs of (**d**) Bi_0.05_Ge_0.99_Te and (**e**) Bi_0.05_Ge_0.99_Te + 0.6 wt.% B_4_C samples.

Figure 1 shows the microstructural and phase characterization of Bi_0.05_Ge_0.99_Te + *x* wt.% B_4_C (*x* = 0, 0.1, 0.4, 0.6, and 1.0) nanocomposites. Electron Probe Microanalysis (EPMA) was utilized to evaluate the elemental homogeneity of the matrix both with and without B_4_C nanoparticles (NPs) incorporation. The pristine Bi_0.05_Ge_0.99_Te sample (Figure 1a) shows a uniform distribution of Bi, Ge, and Te. However, minor dark regions visible in the backscattered electron micrograph correspond to areas with a low characteristic X-ray signal intensity. This reduced signal is most likely caused by surface topography, as local height variations can affect X-ray emission and detection. Upon the addition of 0.6 wt.% B_4_C (Figure 1b), the matrix stoichiometry remains stable, while elemental mapping of Boron (B) and Carbon (C) confirms the presence of the secondary phase. While B_4_C NPs are generally dispersed, some regional concentration variations are observed, likely arising from mechanical processing constraints during synthesis. Chemical composition and elemental distribution were analyzed using Energy Dispersive X-ray Spectroscopy (EDS) via surface area mapping and point analysis mode. The X-ray diffraction (XRD) patterns shown in Figure 1c confirm that all samples are monophasic and index well to the rhombohedral GeTe structure (R3m, PDF#82-8422). No secondary impurity phases were detected, indicating high phase purity across all compositions. Notably, the primary diffraction peaks exhibit slight broadening and a decrease in intensity as B_4_C NPs content increases, which is indicative of grain refinement and strain-induced lattice distortion caused by the NPs inclusions. Scanning Electron Microscopy (SEM) images further reveal the fracture surfaces of the pristine (Figure 1d) and 0.6 wt.% B_4_C-reinforced (Figure 1e) samples. The pristine matrix displays a dense, polycrystalline morphology with clear grain boundaries. The 0.6 wt.% B_4_C nanocomposite retains this fundamental framework; however, the presence of B_4_C NPs at the grain boundaries appears to inhibit grain growth, correlating with the peak broadening observed in the XRD data.

**Figure 2:**
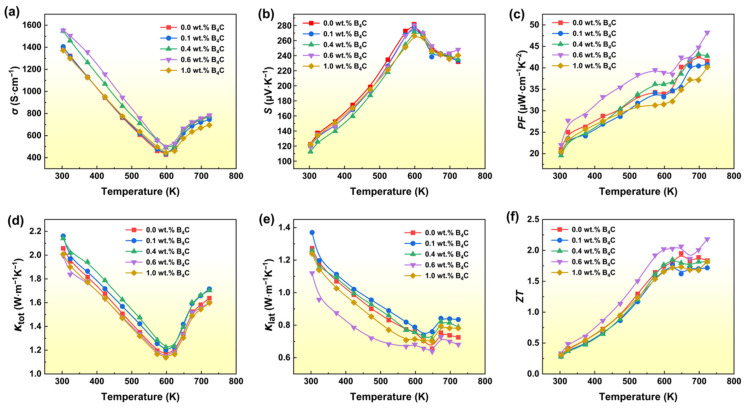
Temperature dependence of TE transport Properties value for GeTe-based nanocomposites: (**a**) Electrical conductivity, (**b**) Seebeck coefficient, (**c**) Power factor, (**d**) Total thermal conductivity, (**e**) Lattice thermal conductivity, (**f**) *ZT*.

Figure 2 shows the temperature-dependent TE transport properties for the Bi_0.05_Ge_0.99_Te + *x* wt.% B_4_C (*x* = 0, 0.1, 0.4, 0.6, and 1.0) nanocomposites. The introduction of B_4_C NPs significantly modulates the electronic transport of the Bi_0.05_Ge_0.99_Te matrix. As shown in Figure 2a,c, the 0.6 wt.% B_4_C composition exhibits the highest electrical conductivity and resulting power factor, identifying it as the optimal concentration for enhanced electronic performance. All samples display degenerate semiconductor behavior, characterized by a decrease in *σ* with increasing temperature up to approximately 600 K (Figure 2a). Conversely, the Seebeck coefficient (*S*) remains largely unaffected or shows a slight enhancement. This behavior is consistent with the energy filtering effect, where the potential barriers created by the B_4_C secondary phase preferentially scatter low-energy carriers, thereby preserving the overall thermopower. Regarding thermal transport, the lattice thermal conductivity decreases with increasing B_4_C content, reaching a minimum value in the 0.6 wt.% B_4_C sample (Figure 2e). This significant reduction in *κ*_lat_ is attributed to intensified phonon scattering at B_4_C/matrix interfaces. The results demonstrate that the strategic dispersion of B_4_C nanoparticles simultaneously improves electrical transport while suppressing thermal conduction. Consequently, Compared to recent reports on GeTe systems, such as the Ge-vacancy suppression reported by Jia et al. and the segmented modules by Pei et al., our B_4_C-reinforced nanocomposites 0.6 wt.% B_4_C exhibit a peak power factor of 48 μW cm^−1^K^−2^ (Figure 2c) and a superior peak *ZT* of 2.2 at 723 K (Figure 2f), underscoring the effectiveness of the nanocomposite strategy in decoupling electronic and thermal transport.

The selection of nanoparticle concentrations was informed by preliminary optimization trials. While higher concentrations (above 1.0 wt.% B_4_C) were found to trigger particle agglomeration and a subsequent decrease in the power factor due to excessive carrier scattering, the current loading levels maximize phonon scattering without compromising electronic transport. This concentration serves as the reinforcement limit for maintaining optimal dispersion in these specific matrix systems. As a results, the Bi_0.05_Ge_0.99_Te + 0.6 wt.% B_4_C nanocomposite was selected as the medium-high temperature segment for the fabrication of the integrated segmented TE single leg device. To validate the reproducibility of these results, we provided a comparison in the (Figure S1) showing the average *ZT* (*ZT*_ave_) across multiple synthesized batches. Additionally, the measurement uncertainty for *ZT* and conversion efficiency is estimated at 7% and 10%, respectively, consistent with international standards for thermoelectric characterization.

**Figure 3:**
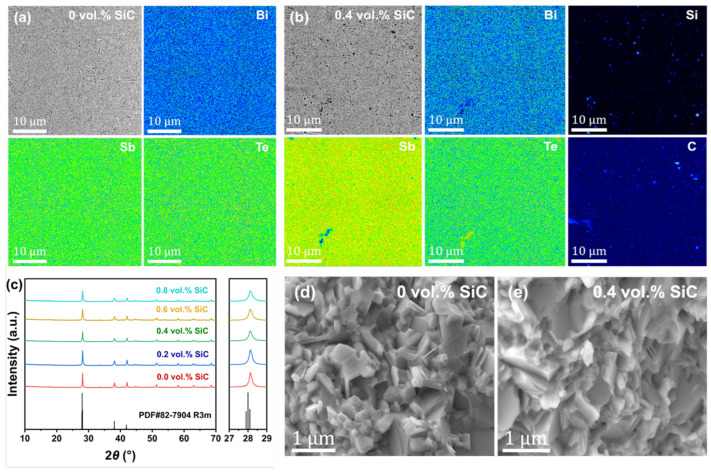
Microstructural characterization of (Bi,Sb)_2_Te_3_-based nanocomposites: (**a**,**b**) EPMA micrograph and EDS mapping of the polished surface of (**a**) Bi_0.4_Sb_1.6_Te_3_ and (**b**) Bi_0.4_Sb_1.6_Te_3_ + 0.4 vol.% SiC samples. (**c**) XRD patterns of Bi_0.4_Sb_1.6_Te_3_ + *x* vol.% SiC nanocomposites (*x* = 0, 0.2, 0.4, 0.6, and 0.8); (**d**,**e**) SEM micrographs of (**d**) Bi_0.4_Sb_1.6_Te_3_ and (**e**) Bi_0.4_Sb_1.6_Te_3_ + 0.4 vol.% SiC samples.

**Figure 4:**
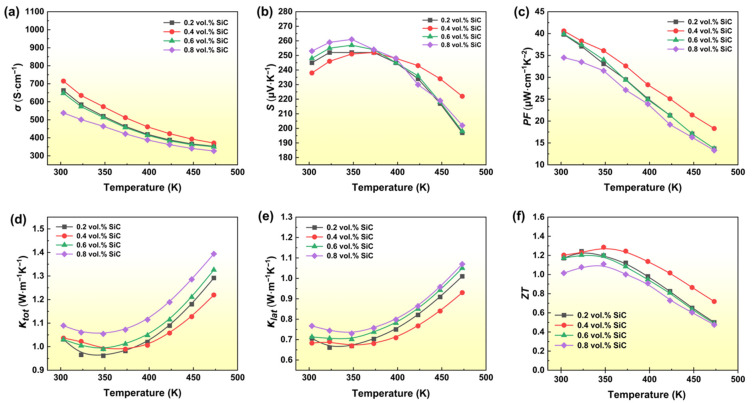
Temperature dependence of TE transport Properties value for (Bi,Sb)_2_Te_3_-based nanocomposites: (**a**) Electrical conductivity, (**b**) Seebeck coefficient, (**c**) Power factor, (**d**) Total thermal conductivity, (**e**) Lattice thermal conductivity, (**f**) *ZT*.

Figure 3 presents the microstructural and phase analysis of Bi_0.4_Sb_1.6_Te_3_ + *x* vol.% SiC (*x* = 0, 0.2, 0.4, 0.6, and 0.8) nanocomposites. Among the various (Bi,Sb)_2_Te_3_ nanocomposites, the 0.4 vol.% SiC sample was selected as the representative optimal composition for detailed microstructural and device-level analysis. In the pristine Bi_0.4_Sb_1.6_Te_3_ sample (Figure 3a), mapping reveals a largely uniform distribution of Bi, Sb, and Te, although minor dark regions suggest subtle localized compositional fluctuations. For the 0.4 vol.% SiC-reinforced nanocomposite (Figure 3b), elemental mapping of Silicon (Si) and Carbon (C) confirms the successful integration of SiC NPs while the host matrix maintains its stoichiometric uniformity. All major diffraction peaks can be indexed to the rhombohedral phase (R3m), which is essentially consistent with the standard card PDF#72-1836 (Figure 3c), and no distinct second-phase diffraction peaks were detected. With increasing SiC content, the diffraction peaks show progressive broadening, primarily attributable to grain refinement. Meanwhile, the observed slight peak shifts toward higher angles indicate a change in lattice parameter, likely resulting from lattice strain induced by the incorporated SiC nanoparticles. These structural modifications, specifically the refined grain structure and induced lattice distortions are anticipated to enhance phonon scattering, thereby suppressing thermal conductivity without compromising electronic transport. SEM images further reveal the fracture surface morphology of the pristine (Figure 3d) and 0.4 vol.% SiC (Figure 3e) samples. The pristine matrix exhibits a characteristic dense, polycrystalline morphology with irregularly shaped grains. The 0.4 vol.% SiC nanocomposite preserves this fundamental granular framework, indicating that the incorporation of SiC at this concentration maintains the structural integrity of the host matrix while providing the necessary interfaces for thermal transport modulation. As revealed by the microstructural analysis, the grain boundaries and strain fields introduced by SiC effectively enhance phonon scattering, leading to a substantial reduction in lattice thermal conductivity (see Figure 4e). Meanwhile, owing to the well-dispersed SiC nanoparticles in the matrix and their relatively low concentration, the obstruction to carrier transport pathways is limited, thereby preserving electrical conductivity (see Figure 4a) and ultimately synergistically improving the *ZT* value.

Figure 4 shows the temperature dependent TE transport properties of Bi_0.4_Sb_1.6_Te_3_ + *x* vol.% SiC (*x* = 0.2, 0.4, 0.6, and 0.8) nanocomposites. As shown in Figure 4a, the electrical conductivity of all samples decreases with increasing temperature in the range of 300–500 K, exhibiting typical metallic behavior of degenerate semiconductors. The Seebeck coefficient undergoes systematic variations with composition and temperature (Figure 4b), indicating that the incorporation of SiC nanoparticles effectively modulates the carrier transport characteristics of the material. The power factor reaches its peak near the temperature range of 350–400 K (Figure 4c). Among them, the 0.4 vol.% SiC sample maintains the optimal *PF* value across the entire tested temperature range, reflecting a good synergy between electrical conductivity and the Seebeck coefficient. Regarding thermal transport, *κ*_lat_ of the 0.4 vol.% SiC composite is most effectively suppressed (Figure 4e), which is mainly attributed to the enhanced multiscale phonon scattering jointly caused by the heterogeneous interfaces, grain refinement, and lattice strain introduced by SiC nanoparticles (see Figure 3). Higher SiC content leads to increased lattice thermal conductivity; as shown in Figure 4e, lattice thermal conductivity increased at concentrations of 0.6 and 0.8 vol.% SiC. Benefiting from the optimized electrical properties and significant reduction in thermal conductivity, the 0.4 vol.% SiC sample achieves the highest *ZT*, peaking at 1.3 at 348 K (Figure 4f). A comparison showing the average *ZT* (*ZT*_ave_) across multiple synthesized batches of (Bi,Sb)_2_Te_3_ nanocomposites is shown in the (Table S2). Based on its optimal and stable *ZT* value, this composition (Bi_0.4_Sb_1.6_Te_3_ + 0.4 vol.% SiC) was selected as the near room temperature segment material for subsequent segmented single-leg TE device. The (Bi,Sb)_2_Te_3_ nanocomposite is expected to possess superior mechanical properties, which may enhance the structural robustness of the device [40].

**Figure 5:**
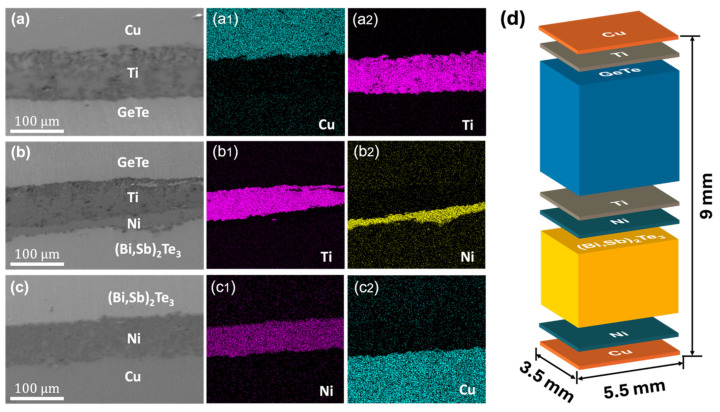
SEM image and EDS mapping of segmented GeTe/(Bi,Sb)_2_Te_3_ single-leg TE device at different interfaces. (**a**,**a1**,**a2**) Cu-Ti-GeTe; (**b**,**b1**,**b2**) GeTe-Ti-Ni(Bi,Sb)_2_Te_3_; (**c**,**c1**,**c2**) (Bi,Sb)_2_Te_3_-Ni-C; (**d**) Schematic diagram of the segmented single-leg device.

Figure 5 presents SEM micrographs and corresponding EDS elemental mapping for the various interfaces within the fabricated segmented GeTe/(Bi,Sb)_2_Te_3_ single-leg TE device. The analysis focuses on three critical transition regions: the Cu/Ti/GeTe junction (Figure 5a), the GeTe/Ti/Ni/(Bi,Sb)_2_Te_3_ segmentation interface (Figure 5b), and the (Bi,Sb)_2_Te_3_/Ni/Cu contact (Figure 5c). EDS mapping results demonstrate highly uniform elemental distributions of Cu, Ti, Ni, Ge, Bi, Sb, and Te across the respective junctions. Specifically, the Ti layer serves as an effective diffusion barrier between the Cu electrode and the GeTe-based segment, while the Ni layer provides chemical stability at the (Bi,Sb)_2_Te_3_ interface. The preservation of these sharp interfaces is essential for maintaining structural integrity and minimizing both parasitic thermal and electrical contact resistances. A schematic illustration of the final integrated device is shown in Figure 5d, detailing the layer-by-layer architecture of the metallization stacks and the active nanocomposites. The segmented single-leg device was fabricated using a one-step Spark Plasma Sintering (SPS) technique to ensure robust interfacial bonding. Initially, a thick Cu layer to serve as the hot-side electrode contact was placed at the bottom of a 10 mm diameter graphite die to serve as the hot-side electrode contact, followed by Ti layer. The GeTe nanocomposite powder was then loaded, followed by a dual-layer Ti/Ni interface layers to prevent interdiffusion between the segments. Subsequently, the (Bi,Sb)_2_Te_3_ nanocomposite powder was added, followed by Ni layer then topped with a final Cu layer for the cold-side electrode contact. The thickness of the metallization layer and Cu layer is ≈100 µm. The entire assembly was sintered at 723 K under a uniaxial pressure of 50 MPa for 5 min. The fabricated single-leg device, measuring 9 mm × 5.5 mm × 3.5 mm, incorporates *p*-type Bi_0.05_Ge_0.99_Te + 0.6 wt.% B_4_C and Bi_0.4_Sb_1.6_Te_3_ + 0.4 vol.% SiC nanocomposites as the medium-high and low-temperature segments, respectively. The photograph of fabricated segmented single-leg device is shown (Figure S3). This strategic material integration leverages the high peak *ZT* values of both segments to maximize efficiency across the entire operating temperature gradient.

**Figure 6:**
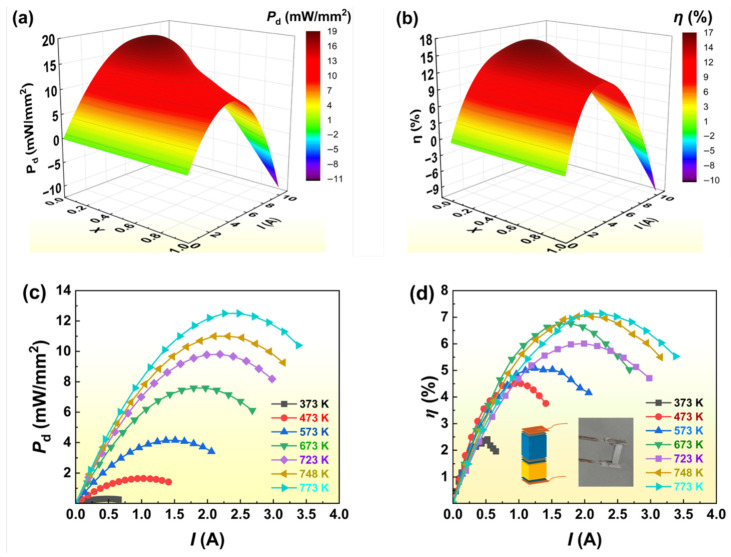
TE performance of the segmented GeTe/(Bi,Sb)_2_Te_3_ Single-leg TE device: (**a**,**b**) Simulated 3D surface plots of (**a**) power density (*P*_d_) and (**b**) efficiency (*η*) versus current (*I*) and segment length ratio (*x*) at *T*_h_ = 723 K and *T*_c_ = 303 K. (**c**,**d**) Measured (**c**) *P*_d_ and (**d**) *η* of the fabricated device from 373 K to 773 K. Insets in (**d**) show the fabricated device schematic and photograph.

Figure 6 presents the TE performance of the segmented GeTe/(Bi,Sb)_2_Te_3_ single-leg device. Finite element simulations were performed to optimize the device configuration under a temperature gradient of *T*_h_ = 723 K and *T*_c_ = 303 K. These simulations targeted the synergy between the high-*ZT* regimes of GeTe-based nanocomposites (500–700 K) and (Bi,Sb)_2_Te_3_-based nanocomposites (300–450 K) by adjusting the segment length ratio (1 − *x*). Numerical contour maps indicate a maximum power density (*P*_d,max_) of 18.5 mW/mm^2^ at *x* = 0.14 and *I* = 7.2 A (Figure 6a), while the peak conversion efficiency (*η*_max_) of 16.9% was identified at *x* = 0.24 and *I* = 5.2 A (Figure 6b). Consequently, a ratio of *x* = 0.24 was selected as the optimal geometric design to maximize efficiency. The performance of the fabricated segmented device was evaluated across a hot-side temperature range of 373–773 K with the cold side maintained at 303 K. As shown in Figure 6c, the experimental power density (*P*_d_) reached 9.81 mW/mm^2^ at 723 K and peaked at 12.5 mW/mm^2^ at 773 K. The achieved power density of (12.5 mW/mm^2^) suggests that these segmented devices could provide a lightweight and efficient solution for recovering waste heat from automotive exhaust systems, where space is a critical constraint. The measured conversion efficiency (*η*) exhibited a significant increase with rising *T*_h_, improving from 2.41% at 373 K to a maximum of 7.14% at 773 K (Figure 6d). The observed shift in peak efficiency toward higher current values at elevated temperatures aligns well with theoretical predictions.

The observed discrepancy between the simulated *η*_max_ (16.9%) and experimental value (7.14%) is primarily attributed to parasitic resistances at the device’s internal interfaces. The simulation model assumes near-zero contact resistance; however, in practice, the multiple junctions (detailed in Figure 5d) introduce cumulative electrical and thermal contact resistances. Specifically, the Ti barrier layer, while effective at suppressing diffusion, contributes to a measurable voltage drop, while the Ni interface at the (Bi,Sb)_2_Te_3_ segment adds thermal boundary resistance that diminishes the actual Δ*T* experienced by the nanocomposite material. Furthermore, heat radiation and conduction losses through the measurement probes in the experimental setup further degrade efficiency. These results successfully validate the segmented design strategy, demonstrating the device’s potential for high-efficiency energy harvesting in high temperature applications.

## 4. Conclusions

This study successfully demonstrated the fabrication of segmented single-leg device based on the enhanced TE performance of GeTe and (Bi,Sb)_2_Te_3_ nanocomposites. By strategically incorporating B_4_C into GeTe and SiC into (Bi,Sb)_2_Te_3_ matrices, lattice thermal conductivity was effectively suppressed through enhanced phonon scattering while maintaining favorable electronic transport, resulting in peak *ZT* values of 2.2 (723 K) and 1.3 (348 K), respectively. These material optimizations provided the foundation for a device architecture capable of maintaining high efficiency across a broad temperature range. Finite element simulations served as a critical framework for device optimization, identifying an ideal geometric segment ratio of 0.24. The experimental validation of the segmented single-leg device, fabricated via a one-step SPS process with effective integration of Ti and Ni metallization barriers, yielded a maximum conversion efficiency of 7.14% and a power density of 12.5 mW/mm^2^ at a hot-side temperature of 773 K. The observed discrepancy between the simulated efficiency (*η*_max_ ≈ 16.9%) and the experimental value (*η*_max_ ≈ 7.14%) arises from several unavoidable practical factors. First, the simulation assumes ideal ohmic contacts with zero resistance, whereas the experimental device involves finite electrical contact resistance at the multiple metallized interfaces, which contributes to internal power dissipation. Second, thermal parasitic losses due to radiative heat loss from the leg surfaces. Finally, although the one-step SPS process minimizes defects, minor interface imperfections and localized carrier scattering at the Ti/Ni barrier junctions can further impede performance. Future study should focus on the implementation of semi-metallic interface materials or silicide barriers, such as FeSi, which have demonstrated the potential to reduce contact resistivity to the µΩ∙cm^2^ level, thereby bridging the gap between theoretical potential and experimental output. This study establishes a robust pathway for the development of high-efficiency thermoelectric devices tailored for high-temperature waste heat recovery applications.

## Data Availability

The original contributions presented in this study are included in the article/Supplementary Materials. Further inquiries can be directed to the corresponding author.

## Supplementary Materials

The following supporting information can be downloaded at: https://www.mdpi.com/article/10.3390/ma19071345/s1, Figure S1: Average thermoelectric figure of merit (*ZT*_ave_). (a) *ZT*_ave_ as s function of B_4_C content (wt.%) and (b) Comparison of *ZT* and *ZT*_ave_ as a function of B_4_C content (wt.%) for Bi_0.05_Ge_0.99_Te + *x* wt.% B_4_C nanocomposites (*x* = 0, 0.1, 0.3, 0.4, 0.6, and 1.0); Figure S2: Average thermoelectric figure of merit (*ZT*_ave_). (a) *ZT*_ave_ as s function of SiC content (vol.%) and (b) Comparision of *ZT* and *ZT*_ave_ as a function of SiC content (vol.%) for Bi_0.4_Sb_1.6_Te_3_ + *x* vol.% SiC nanocomposites (*x* = 0.2, 0.4, 0.5, 0.6, 0.8, 1.0, and 1.5); Figure S3: Fabricated segmented single-leg device photograph; Table S1: Physicochemical and thermal properties of starting raw materials; Table S2: Comparison of this work with similar results reported in the literature.
