# Supplementary material for: Thermoelectric Nanocomposites and Segmented Single-Leg Device Based on GeTe and (Bi,Sb)2Te3
Source: Materials (Basel). 2026 Mar 28;19(7):1345. doi: 10.3390/ma19071345 (PMC13074335; doi:10.3390/ma19071345)
Supplement: Supplementary file 1 [file materials-19-01345-s001.zip › materials-4187793-supplementary.pdf]

Thermoelectric Nanocomposites and Segmented Single-leg Device Based on GeTe and $(Bi,Sb)_2Te_3$

Supplementary Information

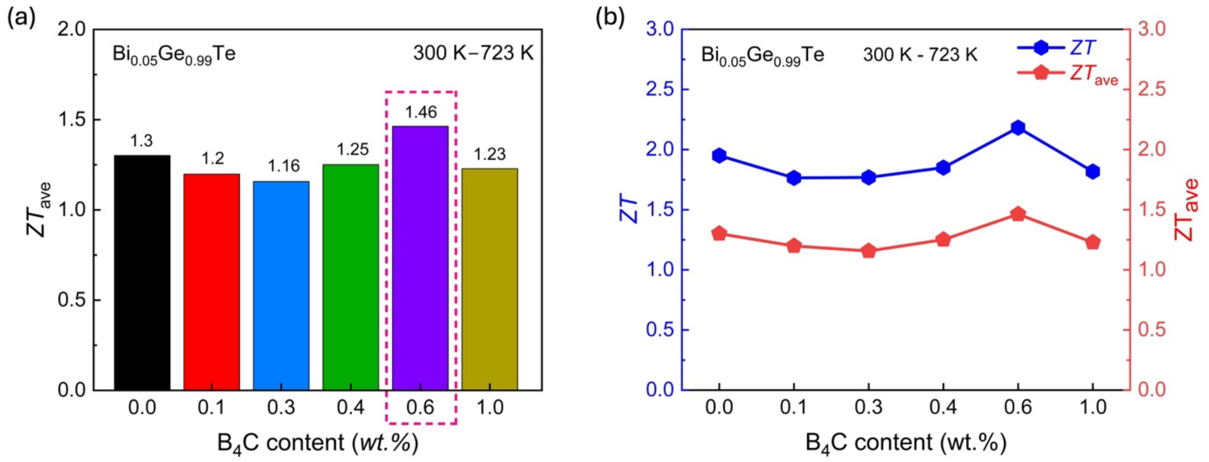

Figure S1: Average thermoelectric figure of merit (ZT_{ave}). a) ZT_{ave} as s function of B_4C content (wt.%) and b) Comparison of ZT and ZT_{ave} as a function of B_4C content (wt.%) for $Bi_{0.05}Ge_{0.99}Te$ + x wt.% B_4C nanocomposites (x = 0, 0.1, 0.3, 0.4, 0.6, and 1.0).

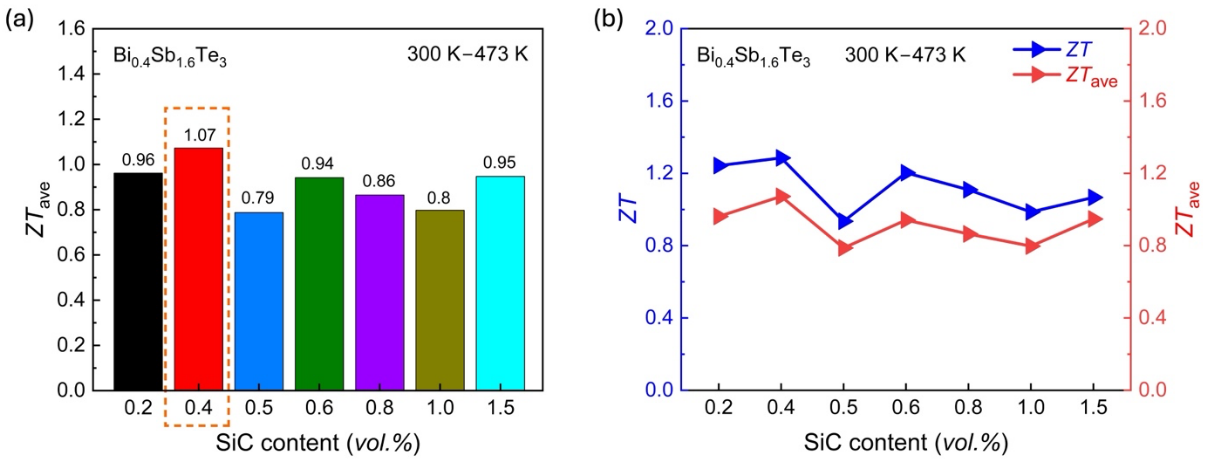

Figure S2: Average thermoelectric figure of merit (ZT_{ave}). a) ZT_{ave} as s function of SiC content (vol.%) and b) Comparision of ZT and ZT_{ave} as a function of SiC content (vol.%) for $Bi_{0.4}Sb_{1.6}Te_3$ + x vol.% SiC nanocomposites (x = 0.2, 0.4, 0.5, 0.6, 0.8, 1.0, and 1.5).

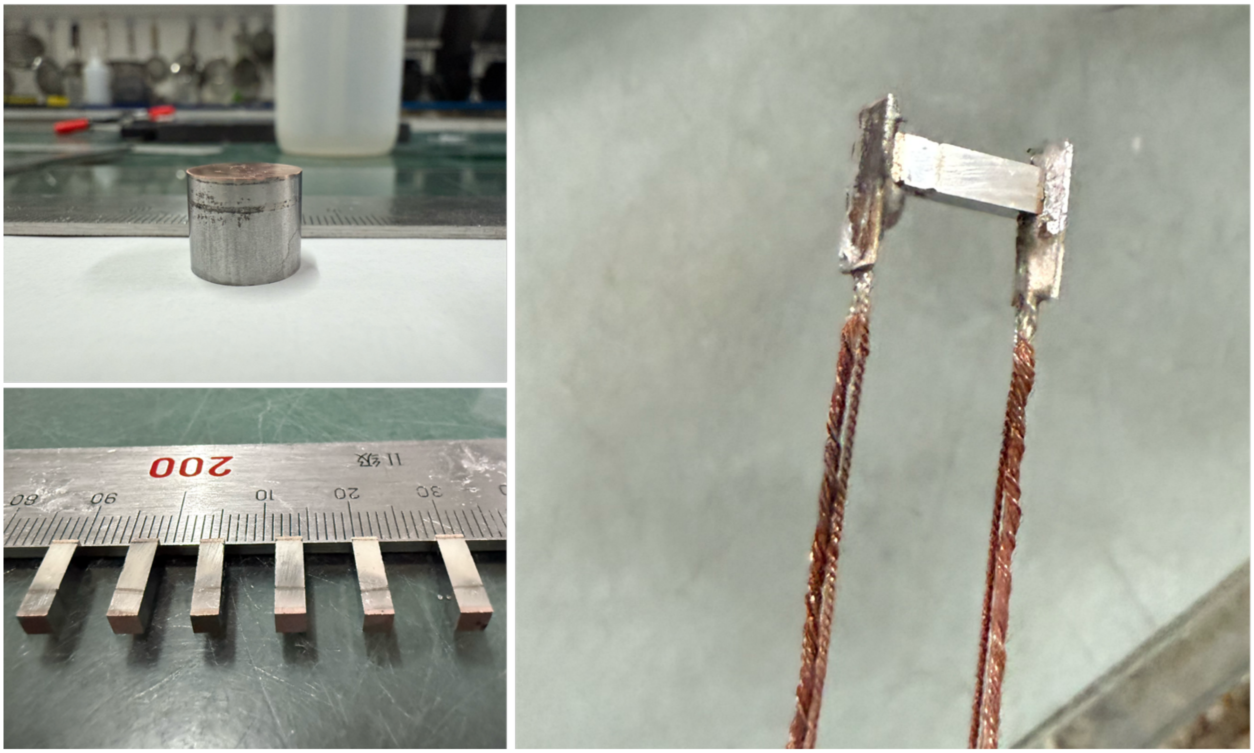

Figure S3: fabricated segmented single-leg device photograph

Table S1. Physicochemical and thermal properties of starting raw materials.

Materials	Purity (%)	Molecular Weight (g/mol)	Avg. Particle Size (µm)	Melting Point (K)	Phase Transition (K)
Ge	99.999	72.63	~75	1211	N/A
Te	99.999	127.60	~75	722	N/A
Bi	99.99	208.98	~75	545	N/A
Sb	99.99	121.76	~75	904	N/A
B_4C	99.5	55.25	~0.5	2623	N/A
SiC	99.5	40.096	~0.05	3100	N/A

Table S2. Comparison of this work with similar results reported in the literature.

**Article Authors**	***ZT* (GeTe)**	***ZT* ($(Bi,Sb)_2Te_3$)**	**Theoretical Power density (mW/mm^2)**	**Fabricated Power density (mW/mm^2)**	**Theoretical Efficiency (%)**	**Fabricated Efficiency (%)**
Cao et al.	1.8	1.1				13.6%
Pei et al.	2.0	1.2		7.45	15.9	9.5
This Work	2.2	1.3	18.5	12.5	16.9	7.14
